# Advances in pediatric flexible bronchoscopy

**DOI:** 10.1007/s12519-025-00967-7

**Published:** 2025-10-04

**Authors:** Shu-Xian Li, Xiao-Fen Tao, Hu-Jun Wu, Fang Jin, Guo-Hong Zhu, Ying-Shuo Wang, Lan-Fang Tang, Zhi-Min Chen, Lei Wu

**Affiliations:** 1 Department of Pulmonology, Children’s Hospital, Zhejiang University School of Medicine, National Clinical Research Center for Child Health, 3333 Binsheng Road, Hangzhou 310052, China; 2 Department of Pediatric Endoscopy, Children’s Hospital, Zhejiang University School of Medicine, National Clinical Research Center for Child Health, 3333 Binsheng Road, Hangzhou 310052, China

Flexible bronchoscopy (FB) is a highly versatile and effective diagnostic and therapeutic tool with a pivotal position in respiratory medicine. Indeed, the flexible bronchoscope, with its capability to visualize both the upper and lower airways, is indispensable for managing neonates, infants, and children with diverse respiratory diseases [1]. Despite the use of guidelines on pediatric flexible bronchoscopy (PFB) from the European Respiratory Society [2], the American Thoracic Society [3] and a Chinese guideline released in 2009 [4] and updated in 2018 [5], considerable variation exists. This is related to the provision of facilities, accessibility of equipment, and implementation. Standards remain to be fully unified.

With the ongoing development of PFB technology, contemporary anesthetic techniques and a growing acknowledgment of the benefits provided by PFB, there has been increased application of this technique in children over the past 4–5 years [6]. Nevertheless, several important points require careful consideration. These are: (1) diverse indications for PFB; (2) preparatory steps prior to personalized bronchoscopy, anesthesia, and bronchoalveolar lavage (BAL) in accordance with contemporary practice; (3) striking a balance between safety and therapeutic effectiveness; and (4) principal challenges for future development. In this editorial, we discuss these important points with an emphasis on advances, improving understanding, implementation, and advocacy for PFB.

## History of pediatric flexible bronchoscopy

Clinical application of bronchoscopy originated in 1897 with Killian’s successful extraction of a pork bone from the right main bronchus [7]. Flexible fiberoptic bronchoscopy was conceived later by Shigeto Ikeda in 1967; it was initially limited to use in adults [8]. Pediatric fiberoptic bronchoscopy was pioneered by Drs. Robert Wood and Robert Fink in 1978 [9]. However, it lacked a working channel and primarily served the purpose of visual examination [9]. A significant advance occurred in 1980 with the introduction of a prototype flexible bronchoscope featuring a 3.5 mm outer diameter and a 1.2 mm working channel, which highlighted fundamental therapeutic capabilities (e.g., BAL) for pediatric patients across all age groups [10]. Since 2000, electronic bronchoscopes (e.g., Olympus BF-P260F) have gradually replaced traditional fiberoptic scopes, offering high-definition imaging, video recording functionalities, and a safer operational experience. In 2002, the introduction of the Olympus BF260 series of flexible bronchoscopes, known as “hybrid-type” bronchoscopes, incorporated standard fiberoptic equipment with newer video chip technology. This allowed for smaller outer diameters, larger working channels, and improved visual resolution [6]. With an outer diameter of 4.0 mm and a working channel of 2.0 mm, the “slim” Olympus BF-P260F bronchoscope provided pediatric pulmonologists with superior suction capability and broadened access to instruments that were previously exclusive to their adult counterparts. This included protected cytology brushes, standard biopsy forceps, and foreign body retrieval baskets [6]. In 2004, the “ultraslim” Olympus BF-XP260F bronchoscope, featuring a 2.8 mm outer diameter and a 1.2 mm working channel, enabled diagnostic and therapeutic procedures to be performed in small, premature infants [6].

Evolution of bronchoscopy from the early limitations of rigid scopes to the revolutionary adoption of fiberoptic technology, and now to state-of-the-art miniaturization of electronic scopes, spans more than 100 years [11]. This evolution in size reduction runs alongside advances in anesthetic techniques, the establishment of standardized procedural protocols, and the fostering of multidisciplinary collaboration [11].

## Indications and contraindications for pediatric flexible bronchoscopy

The decision to pursue PFB for diagnostic and/or therapeutic purposes is invariably based on a careful assessment of each individual case, taking into account medical history of the patient, physical examination, and results from previous diagnostic evaluations. Indications for PFB are summarized in Table 1 [2–5]. In recent years, substantial progress in the application of innovative tools designed for endobronchial procedures and image-guided tissue sampling has drastically expanded the scope of PFB. Indeed, this has led to the introduction of two new concepts: advanced diagnostic bronchoscopy and interventional bronchoscopy [12–14]. Specifically, advanced diagnostic bronchoscopy has encompassed an array of sophisticated techniques, notably endobronchial ultrasound (EBUS)-guided transbronchial lung and lymph node biopsy, computed tomography (CT) navigation, and use of robotic technology [14]. Advanced interventional bronchoscopy refers to therapeutic procedures that include laser ablation, cryotherapy, electrocautery, argon plasma coagulation, tissue debulking, balloon dilation, stent placement, injection, and foreign body removal [14]. Applications of advanced diagnostic and interventional bronchoscopy are outlined in Table 2 [12–14].

**Table 1:** Indications for pediatric flexible bronchoscopy

Diagnostic use
Airway obstruction
Inspiratory stridor or abnormal respiratory sounds
Persistent or recurrent expiratory wheezing
Radiological abnormalities
Tracheal and/or bronchopulmonary dysplasia and/or malformation
Persistent atelectasis
Recurrent or persistent pulmonary consolidation
Atypical or unexplained pulmonary infiltrates
Localized emphysema
Diffuse lung disease
Abnormal development of blood vessels, lymphatic vessels and esophagus
Pulmonary mass lesions
Airway and mediastinal occupying lesions
Chronic cough of unknown cause
Suspected structural anomalies (e.g., congenital, anatomical or acquired)
Suspected airway foreign body aspiration
Recurrent respiratory tract infection
Hemoptysis or pulmonary hemorrhage
Difficult weaning
Lung allograft or artificial airway monitoring
Obstructive sleep apnea
Therapeutic use
Restoring airway patency (e.g., mucus plug clearance and foreign body removal)
Pulmonary alveolar proteinosis (e.g., alveolar proteinosis or lipid pneumonia)
Others
Bronchoalveolar lavage
Bronchial mucosal lesions; brushing and biopsy
Transbronchial lung biopsy
Local drug administration
Interventional therapy
Guiding endotracheal intubation

**Table 2:** Applications of advanced diagnostic and interventional bronchoscopy

Application	Bronchoscopic technologies
Advanced diagnostic bronchoscopy	
Peripheral lesions	Radial endobronchial ultrasound
	Ultrathin bronchoscopes
	Virtual bronchoscopy
	Navigation and robotic-assisted bronchoscopy
Mediastinal lesions	Endobronchial ultrasound with transbronchial needle aspiration/fine needle biopsy
	Endobronchial ultrasound guided intranodal forcep biopsy
Diffuse parenchymal lung disease	Cryobiopsy
Advanced therapeutic bronchoscopy	
Central airway obstruction	Bronchoplasty and mechanical debridement
	Thermal ablative therapies (including laser, contact electrocautery or argon plasma)
	Cryoablative therapies
	Balloon dilation
	Airway stents

Contraindications for PFB are primarily influenced by operator skill and equipment availability. Relative contraindications for PFB include severe cardiopulmonary dysfunction, life-threatening arrhythmias (e.g., atrial fibrillation, ventricular fibrillation, flutter, and third-degree atrioventricular block), advanced pulmonary hypertension, hyperpyrexia, active/impending massive hemoptysis, significant bleeding disorders, severe malnutrition, and an inability to tolerate surgical interventions [2–5]. Even when faced with challenges such as cardiovascular instability, pulmonary hypertension, coagulopathy, severe hypoxemia or respiratory failure, appropriate measures can be implemented for the safe use of PFB where this is considered necessary [2–5]. Ultimately, both benefits and risks should be individually assessed. We consider it prudent to avoid PFB when the risks outweigh the potential benefits.

## Preoperative preparation for pediatric flexible bronchoscopy

The key points of preoperative preparation for PFB are listed in Table 3 [4, 5, 15]. A thorough preoperative evaluation (e.g., disease severity, optimal timing for surgery, anesthetic protocol, and emergency response plan) prior to PFB is crucial to guarantee successful execution of the procedure and reduce the risk of complications.

**Table 3:** Key points for preoperative preparation for pediatric flexible bronchoscopy

Obtaining a complete medical history and performing a thorough physical examination, focusing on the suspected underlying pathology
Identification and management of comorbidities that may potentially influence the administration of anesthesia or sedation, complicate the procedure or prolong postoperative recovery, including:
Patients with a history of bronchial hyperresponsiveness may benefit from inhalation of glucocorticoids (e.g., budesonide suspension 2 mL/time, administered every 6–8 h) and bronchodilators before PFB
In cases of severe asthma, the administration of intravenous glucocorticoids and inhalation of bronchodilators is crucial, with bronchoscopy procedures being postponed until the condition of the patient is stabilized
In non-emergency scenarios, bronchoscopic interventional therapy for children with tracheobronchial tuberculosis should be performed 2 wk after initiating systemic anti-tuberculosis chemotherapy to prevent the spread of infection
Convulsions and seizures must be adequately managed prior to PFB
Psychological support to alleviate patient anxiety
Required routine pre-procedure laboratory evaluations, including:
Routine blood examination
Assessment of coagulation status
Serological screening for hepatitis B and C, human immunodeficiency virus and syphilis
Liver and kidney function assessments
Radiographic imaging (e.g., X-ray, chest computed tomography)
Electrocardiogram
Written informed consent, including:
Parents or guardians, along with age-appropriate children should be fully informed regarding the PFB procedure, including its purpose, alternative methods, potential complications and associated risks of the procedure or anesthesia
The anesthesiologist should secure a separate signed consent for anesthesia and inquire about any drug allergies for patients scheduled for general anesthesia
Fasting durations adjusted based on the different timeframes required for gastric emptying^a^, e.g.,
2 h for clear liquids
4 h for breast milk
6 h for milk, formula milk and starch-based solids
8 h for high-fat-based solids

*PFB* pediatric flexible bronchoscopy. ^a^Infants and newborns, due to their limited glycogen reserves, should receive intravenous infusions of sugar-containing fluids after 2 hours of fasting to prevent hypoglycemia and dehydration

## Anesthesia

Selection of an appropriate anesthetic is crucial for the smooth implementation of PFB. This is influenced by several clinical factors, including disease characteristics, nature and demands of the procedure, patient safety and comfort, availability of requisite equipment and skilled professionals, and adequate conditions for therapeutic or diagnostic PFB. The most commonly employed anesthetic protocols include topical, local combined with conscious sedation, and general anesthesia.

In response to a growing demand for humanistic care, local surface anesthesia is seldom used in isolation. Except in instances involving children where it is crucial to monitor the dynamic airway characteristics of the airway, a combination of local surface anesthesia and conscious sedation or general anesthesia is generally preferred in the majority of cases.

The anesthetic technique of “local anesthesia while proceeding” coupled with conscious sedation for bronchoscopy is straightforward to implement and does not require the participation of anesthesiologists. The incidence of peri-procedure-related respiratory depression is minimal, rendering this approach ideal for brief interventions such as BAL. However, this approach may provoke adverse events such as limb movement and coughing, especially in those who experience insufficient sedation.

General anesthesia typically administered by anesthesiologists enables patients to withstand longer procedures and significantly reduces the likelihood of endoscopic complications (e.g., bleeding, injury, and pneumothorax). With the involvement of anesthesiologists, the anesthetic plan can be rapidly modified in response to changing circumstances. For instance, if a bronchial foreign body is not removed using PFB, anesthesiologists can swiftly deepen anesthesia to facilitate extraction via rigid bronchoscopy. General anesthesia markedly improves the acceptability of PFB among children and their caregivers [16, 17]. Based on different ventilatory methods, general anesthesia is classified into intravenous combined anesthesia, laryngeal mask airway anesthesia, and tracheal intubation anesthesia (Table 4).

**Table 4:** Advantages and disadvantages of different general anesthesia regimens

Regimen	Application scenarios	Advantages	Disadvantages
Intravenous combined	Bronchoscopies with shorter procedural times and less stimulation, e.g., routine airway assessment, bronchoalveolar lavage, brush biopsy	Maintain spontaneous breathing during examination	Limited level of sedation and unable to fully cooperate with the bronchoscopic procedure in some patients
		Relatively small dose of anesthetic drugs	Risk of respiratory depression requiring close monitoring
		Effectively inhibits the cough reflex and reduces discomfort	
		Independence from artificial airways, enabling concurrent visualization of the upper respiratory tract, glottis, piriform sinus and other structures	
		Swiftness, time-efficiency and cost-effectiveness, by obviating the need for intubation and extubation	
		Effectiveness of specialist training, by alleviating the complexity of bronchoscopy operation	
Laryngeal mask airway ventilation	Procedures of longer duration and with greater stimulation, e.g., examination of complex lesions	Completely unconscious during the examination	Risks of laryngeal mask placement failure or inadequate ventilation
	Procedures in the laryngeal area and below, e.g., tracheal stenosis, especially those with upper segmental stenosis	Easy to operate and causes minimal airway irritation, facilitating spontaneous breathing	Deeper level of anesthesia
		Reduce the occurrence of adverse reactions, e.g., procedural airway and laryngeal spasms	Laryngeal and bronchial spasms during mask insertion
		Emerges as a more appropriate and recommended approach when bronchoscopy is not feasible through a tracheal tube	
Intubation	Complex and high-risk bronchoscopic procedures, e.g., complex interventional bronchoscopy and severe airway stenosis	Providing stable airway ventilation with a low risk of leakage	Tracheal intubation requires skill and experience
		Better control of airway secretions and prevention of aspiration	Causing airway stimulation during intubation, which may lead to adverse reactions, e.g., laryngeal or bronchial spasms, or cardiovascular events
		Swift transition to one-lung ventilation in the event of massive bleeding	Risks of intubation failure or inadequate ventilation
			Deeper level of anesthesia and intubation, obscuring dynamic airway changes
			Airway injury or laryngeal edema after intubation
			Poor upper airway and proximal tracheal view

## Bronchoalveolar lavage

BAL is a versatile tool for the diagnosis, prognosis evaluation, and clinical treatment of various respiratory diseases. These include pulmonary infections, hypersensitivity pneumonia, asthma, diffuse lung disease, as well as opportunistic infections in immunocompromised patients [18]. For most clinical applications, the flexible bronchoscope is gently wedged into the target bronchus, and normal saline at 37 °C is sequentially instilled through the working channel of the bronchoscope. The recommended volume for BAL in pediatric patients is 1 mL/kg per aliquot, with a maximum of 20 mL for each aliquot. The total lavage volume should not exceed 5–10 mL/kg, to be administered in 3–4 consecutive aliquots. BAL fluid (BALF) is gently recovered using a suction device with a selected negative pressure to avoid airway collapse; the minimal total volume retrieved is recommended to be ≥ 40% of the instilled volume [4, 19].

Although the cellular composition of BALF in healthy individuals varies in different studies [4, 19, 20], both domestic and international guidelines describe the proportion of cells in BALF as follows: lymphocytes 10%–15%, neutrophils ≤ 3%, eosinophils ≤ 1%, and alveolar macrophages (Am) > 85% [5, 21]. Atypical shifts in the ratios of cellular components in BALF provide critical insights in the diagnosis of pulmonary disorders [4, 21, 22] (Table 5). Moreover, BALF is more suitable than sputum for the detection of specific microbiological infections, as this tends to be less contaminated by oral bacteria during collection [23]. Identification of pathogens in BALF provides critical information on respiratory tract infection and enables both precise antibiotic therapy and assists in prognostic assessment [24, 25]. In addition, BAL serves as a potent therapeutic intervention for the removal of pathogenic agents (e.g., lipids [26], proteinaceous substances [27], and mucus [28, 29]) and inflammatory mediators from the alveoli in a spectrum of pediatric pulmonary disorders (e.g., exogenous lipid pneumonia [26], pulmonary alveolar proteinosis [27], and refractory pneumonia [28, 29]). Collectively, this can mitigate inflammatory responses, enhance pulmonary ventilation, alleviate clinical symptoms, and improve prognosis.

**Table 5:** Disorders associated with abnormal BALF cell compositions

Cellular composition of BALF	Lymphocytes	Neutrophils		Eosinophils	Macrophages
Healthy individuals	10%–15%	≤ 3%		≤ 1%	> 85%
Hypersensitivity pneumonitis	↑, 30%–70%				
Eosinophilic pneumonia, asthma, allergic bronchitis				↑, 20%–95%	
Idiopathic pulmonary fibrosis, connective tissue diseases		↑			↓
Diffuse alveolar hemorrhage and hemosiderosis					↑, contains free red blood cells loaded with hemosiderin or phagocytosed cells
Alveolar proteinosis					↑, displays a swollen foamy appearance

*BALF* bronchoalveolar lavage fluid, *↑* increase, *↓* decrease

## Advanced diagnostic bronchoscopy

Mediastinal lymphadenopathy poses a considerable clinical challenge in pediatric patients; however, the emergence of transbronchial needle aspiration (TBNA) improves the diagnostic efficacy of FB (Table 6). The youngest patient documented to have undergone conventional TBNA (c-TBNA) with a flexible bronchoscope was only 9 months old [30]. Although c-TBNA is safer than classic endoscopic techniques (e.g., mediastinoscopy, video-assisted thoracoscopic surgery, and thoracotomy), it is a “blind” procedure that is restricted to sampling larger subcarinal/right paratracheal lymph nodes. EUBS-TBNA which typically employs linear probe ultrasound for real-time TBNA presents a minimally invasive and safe alternative. In institutions with experienced bronchoscopy and anesthesia teams, EBUS-TBNA can be employed for sampling mediastinal or hilar lesions and diagnosis of a variety of conditions, such as leukemia, lymphoma, sarcoidosis, tuberculosis, and other infections [31–33]. Current literature reveals that the youngest patient to receive EBUS-TBNA was 6 years old, using an Olympus BF-UC160F with a 6.9 mm outer diameter [34]. Therefore, EBUS-TBNA with a 6.3 mm outer diameter (such as the Fujifilm EB-530US or the Olympus BF-UC290F) could theoretically be considered for even younger patients.

**Table 6:** The application and characteristics of advanced diagnostic bronchoscopy

Technique	Application scope	Advantages	Disadvantages
c-TBNA	Mediastinal and hilar lymph node biopsies	No need for ultrasound devices	Operator-dependent
		Lower cost	High risk of blind puncture
		Relatively simple procedure	Rarely used in children, as smaller airways in children increase procedural difficulty and complication risks
		Suitable for resource-limited medical institutions	
		Minimizes the need for more invasive techniques (e.g., mediastinoscopy)	
EBUS-TBNA	Mediastinal and hilar lymph node biopsies, especially for older individuals with sufficiently large airways and lymph nodes located near the trachea	Allowing for the real-time sampling of small mediastinal nodes that are otherwise inaccessible by other methods and effectively targeting smaller lymph nodes located in the challenging areas (e.g., left paratracheal area)	Expensive equipment
		Facilitating precise visualization of the relationship between the needle, the lesion and surrounding blood vessels through ultrasound images	Complex procedure
		Increasingly used in children, mainly for older children	Requires sedation or anesthesia in children
		Improving specimen acquisition, enhance diagnostic accuracy, and minimize complications as well as the need for more invasive surgical interventions	Smaller airways increase procedural difficulty
EUS-B-FNA	Mediastinal lesion biopsies, especially for lymph nodes neighboring the esophagus or in younger patients with narrower airways	Capable of assessing lesions adjacent to the esophagus or located in the lower posterior mediastinal region (e.g., paraesophageal, vertebral and paravertebral lesions)	Requires endoscopy and ultrasound equipment
		Capable of applying in situations where a transbronchial approach is contraindicated due to the patient’s condition	High cost
		Not affected by airway conditions, especially recommended in the following scenarios Patients with respiratory failure experiencing hypoxemia or hypercapnia who are not suitable for sedatives Patients without hypoxemia but with enlarged mediastinal lesions compressing the tracheal, complicating EBUS-TBNA evaluation Patients with excessive coughing who are unable to undergo EBUS-TBNA	Limited use in children due to complex procedures
		Fewer instances of oxygen desaturation and allowing for lower sedative dosages while maintaining patient comfort	
rpEBUS	Peripheral lung lesions, especially peripheral pulmonary nodules	Capable of assessing peripheral lung lesions	Expensive equipment
		Combining navigation technology improves diagnostic rate	Requiring virtual navigation technology
		Minimally invasive	Complex procedure
		Fewer complications	Rarely used in children due to smaller airways and increased procedural difficulty

Since the diameter of available EBUS scopes (usually 6.9–7.4 mm) exceeds those of conventionally used pediatric flexible bronchoscopes (typically 2.8–4.2 mm), performing EBUS-TBNA poses challenges in younger children with smaller tracheas [35]. As a viable alternative, endoscopic ultrasound with bronchoscope-guided fine needle aspiration (EUS-B-FNA) [36] has gained support for its ability to assess lesions that are inaccessible via the airway and serves as the exclusive approach for small children. The youngest reported age for the application of EUS-B-FNA is currently 20 months [37].

When choosing between EBUS-TBNA and EUS-B-FNA, several factors should be considered; these include: lymph node location, patient age, and airway diameter [31]. When both methods are feasible for target lymph node access, the decision should harmonize patient-specific situations and operator proficiency to guarantee a safe and effective diagnostic outcome [38].

Radial probe EBUS (rpEBUS) employs a flexible catheter housing a rotating ultrasound transducer that generates a comprehensive 360° “radial” ultrasound image [39]. Direct contact rpEBUS probes, featuring outer diameters of 1.7 to 2.5 mm, are capable of delivering high-resolution images at frequencies between 12 and 30 MHz and can be advanced through the working channel of a pediatric-size bronchoscope for the identification and assessment of peripheral lung lesions [40]. Bouso et al. were pioneers in the application of rpEBUS-guided transbronchial biopsy in children where they showcased its practicality, safety, and increased diagnostic tissue yield in immunocompromised children with chest radiographic opacities [41].

Despite the widespread application of c-TBNA, EBUS-TBNA, EUS-B-FNA, and rpEBUS in adults, there exists a dearth of literature regarding use and efficacy in pediatric populations [31, 38–41]. While collaboration with adult pulmonologists offers numerous opportunities for the evolving discipline of pediatric interventional pulmonology (such as improved transfer of skills and expertise), it is essential to acknowledge the considerable disparities in disease patterns, anatomical factors, and treatment methodologies between adult and pediatric populations. To ensure the field flourishes, there is a pressing need to strengthen awareness of, and training, pediatric interventional bronchoscopy through global cooperation and the development of educational courses [42].

## Advanced interventional bronchoscopy

### Airway foreign body removal

Foreign body aspiration (FBA) is a common cause of pediatric emergency visits and is among the leading causes of endobronchial obstructions encountered in children [43, 44]. Rigid bronchoscopy is the preferred diagnostic and therapeutic approach for pediatric FBA [45]. However, its application may be limited in a number of circumstances, including foreign bodies in the bilateral upper lobe, small foreign objects embedded in narrow/distal airways, anatomical anomalies impeding the passage of rigid bronchoscopes, and suspected residual foreign body fragments following retrieval via rigid bronchoscopy. Our previous study suggested PFB could be a viable first-line option for extracting airway foreign bodies by skilled personnel with optimal equipment (e.g., biopsy forceps, grasping forceps, and wire baskets) [46], as shown in other studies [47, 48].

Use of PFB in the exploration of suspected airway foreign bodies is strongly advocated in the following scenarios [49–51]: (1) absent clinical symptoms and normal chest radiographs or CT scans following a choking episode; (2) suspicion of a residual foreign body after an attempted removal via rigid bronchoscopy [52]; and (3) symptoms (e.g., stridor, noisy breathing, dysphonia, and new onset/recurrent/persistent wheezing) with abnormal chest X-ray findings and unilateral reduced aeration upon auscultation, even in the absence of a clear history of aspiration. The application of this technique may occasionally fall short in situations involving excessively large, sharp, or obstructive foreign objects that have precipitated unilateral atelectasis. This could result in the foreign body getting stuck at the glottis or blocking the contralateral airway with life-threatening sequelae (e.g., asphyxia) [53]. In these situations, rigid bronchoscopy offers a more expeditious means of retrieving the dislodged object and mitigating the risk of asphyxia [53]. Immediate availability of a rigid bronchoscope is needed when flexible techniques prove inadequate.

Extraction of intricate bronchial foreign bodies requires the combined efforts of otolaryngology and respiratory departments, centering on the synchronized operation of both flexible and rigid bronchoscopes [53]. In instances where the foreign body is firmly impacted, laser ablation may be used via a flexible bronchoscope to fragment the object, allowing for its subsequent retrieval with a rigid bronchoscope [54]. For patients with an endotracheal tube in situ, a flexible bronchoscope can be introduced through the tube to evaluate the foreign body and guide subsequent actions. These include simultaneous extraction of both the tube and the foreign body or first withdrawing the tube followed by the use of a rigid bronchoscope for removal.

### Tracheal intubation using flexible bronchoscopy

The estimated incidence of difficult tracheal intubation in the pediatric population ranges from 0.9% to 5.8%, with infants and neonates experiencing higher rates [55, 56]. An increase in the number of tracheal intubation attempts in children is associated with a greater risk of complications, such as laryngeal edema, hypoxemia, and even cardiac arrest [56, 57]. Employing a flexible bronchoscope as a steerable stylet to assist with tracheal intubation is regarded as a safe, effective, and preferred method for managing anticipated and unanticipated difficult airway [58]. This is especially the case for children with laryngeal masses, diminutive mandibles, Pierre Robin sequence, airway deformities, restricted mouth openings, obesity, cervical spine injuries, cervical ankylosis, ankylosing spondylitis, airway compression from cervical and mediastinal masses, mandibular fractures, cervical hemangiomas, facial injuries, and trismus [58, 59].

### Flexible bronchoscopic interventional techniques

Despite the rapid expansion of bronchoscopic interventions in adults, these procedures are less commonly performed in pediatric patients for several reasons. These include narrow airway size, lack of appropriately sized equipment, requirement for general anesthesia, absence of pediatric interventionalists or limited technique knowledge, and shortage of specialized expertise.

Bronchoscopic laser ablation, with its high energy concentration and limited thermal radiation, efficiently eliminates caseous necrotic debris and granulation tissue. This facilitates lung re-expansion while minimizing the likelihood of iatrogenic scar stenosis, hemorrhage, and luminal occlusion [60–62]. Compared to electrocoagulation and argon plasma coagulation probes, laser fibers possess a slimmer diameter allowing them to traverse the 1.2 mm working channel of a bronchoscope making this applicable to all pediatric age groups.

Balloon dilatation represents a remarkably effective and minimally invasive approach for the treatment of airway stenoses, including those resulting from congenital anomalies, complications following post-intubation, sequelae from tuberculosis, surgical scarring, and to obtain endobronchial occlusion in cases of bronchopleural fistula or massive hemoptysis [63]. While balloon dilatation is broadly considered a safe and well-tolerated procedure, the risk of post-procedure restenosis necessitates repeated bronchoscopic monitoring and additional dilatation. In some instances, laser ablation may be employed for radially dissecting thick, mature scars using flexible bronchoscopes prior to the balloon dilation procedure [63].

The evolution of cryoprobes, now available in sizes as small as 1.1 mm, has made cryotherapy a feasible therapeutic option for addressing pediatric airway pathology in children of all ages [64]. Cryotherapy employing cryogens such as nitrogen, nitrous oxide, and carbon oxide enables the probe tip to achieve a cooling temperature of approximately – 50 ℃ to facilitate procedures including biopsy, restoration of airway patency, and foreign body retrieval [64, 65].

Electrocautery utilizes an alternating high-frequency electric current transmitted through a probe to generate heat, enabling cutting, coagulation, and/or vaporization of tissue. Its effectiveness in managing both malignant and benign central airway obstruction with pathologies such as granulation tissues, papillomas, hamartomas, and tracheal lobular capillary hemangioma has been well established [66–68].

The advanced therapeutic techniques described above are increasingly utilized in pediatric patients with a widening array of applications (e.g., tuberculosis, complex airway foreign body removal, acquired subglottic stenosis, and central airway obstruction) in well-equipped centers by well-trained and experienced practitioners [61, 62, 69–72]. These multimodal therapeutic bronchoscopic airway interventions provide a safe, effective, minimally invasive, time-efficient, cost-saving strategy for tackling intricate airway issues, either individually or in combination [62, 70]. While well-established criteria for these advanced therapeutic approaches are still lacking, their selection depends on the size, shape, location, and characteristics of the airway lesion, physician preference and expertise, and the availability of required equipment.

## Safety concerns and strategies

Common complications associated with PFB include anesthetic drug allergies, decreased blood oxygen saturation or hypoxia, asphyxia, arrhythmia, laryngospasm or bronchospasm, epistaxis, hemoptysis, infection, fever, pneumothorax, as well as mediastinal and subcutaneous emphysema [4, 5]. The most common causes of complications stem from inadequate bronchoscopic technique and lack of experience [73], insufficient preoperative evaluation (comorbidities) [74, 75], selection of an inappropriate bronchoscope model for the patient’s needs, improper anesthetic sedation, insufficient piped oxygen supply, ineffective infection control, inadequate perioperative nebulized medication therapy, and difficulties in establishing effective intravenous access. Life-threatening adverse events primarily arise from anesthetic drug overdose, inadequate monitoring, and inadequate sedation. Major risk factors include upper airway pathology, persistent radiographic abnormalities, oxygen dependency, and body weight < 10 kg [76]. Desaturation is the most frequent complication during the procedure, closely followed by epistaxis. Cough is the most prevalent post-bronchoscopy complication, succeeded by fever [77, 78].

The key to minimizing complications in PFB lies in comprehensive preoperative evaluation and preparation, standardized intraoperative procedures with real-time monitoring, close postoperative observation and timely intervention, along with multidisciplinary teamwork and well-equipped facilities [5]. Throughout the procedure, it is vital to continuously monitor vital signs to ensure the oxygen saturation exceeds 95%. If this dips below 85% in conjunction with an abnormal heart rate, the procedure should be immediately halted and appropriate intervention initiated. Continuous postoperative monitoring for at least 2 hours is required to intervene where dyspnea, hemoptysis, and fever occur. Owing to the influence of anesthesia, children are advised to abstain from food and drink for a minimum of 2-hour post-procedure. For complex cases, multidisciplinary cooperation and personalized treatment strategies play a crucial role in reducing complications and improving therapeutic outcomes [72, 79–81].

## Challenges and prospects

PFB has evolved beyond a purely diagnostic tool to become an indispensable procedure in modern pediatric respiratory medicine. Its practical relevance is undeniable, offering minimally invasive access for the identification of intricate airway anomalies, infectious etiologies, and FBA aspiration, while increasingly enabling crucial therapeutic interventions. The ability to perform PFB under conscious sedation in a wide range of pediatric patients further emphasizes its practical application and patient-friendly characteristics, allowing dynamic assessment and targeted sampling. Nevertheless, there are challenges that require focus. These include optimizing procedural protocols and ensuring safety for the youngest and most critically ill patients. Moreover, improving diagnostic yields for elusive conditions through advanced ancillary techniques, establishing robust standardized training and credentialing pathways globally to guarantee consistent quality and safety, and tackling resource disparities that restrict access are additional challenges. Future endeavors should be concentrated on: (1) advancing technological innovation to develop smaller, higher resolution scopes and more sensitive point-of-care diagnostic tools; (2) implementing and evaluating structured, competency-based training programs to bridge the training gap; (3) fostering collaborative, multi-center research to yield robust pediatric-specific evidence concerning outcomes, techniques, and novel applications; and (4) identifying and addressing barriers to equitable access to PFB expertise. Addressing these challenges and striving in this direction is imperative to unlocking the full potential of PFB, ensuring all children benefit from its diagnostic accuracy, therapeutic capabilities, and minimally invasive approach.

## Data Availability

Data sharing is not applicable to this article as no datasets were generated or analyzed.
